# The Prognosis of Axial vs. Extra-Axial Lesions on Functional Status After Traumatic Brain Injury

**DOI:** 10.3389/fneur.2019.01167

**Published:** 2019-11-01

**Authors:** Nhung Quach, Jacqueline Do, Benjamin Dirlikov, Reza Ehsanian, Elizabeth C. Pasipanodya, Arshad Ali, Thao Duong

**Affiliations:** ^1^Rehabilitation Research Center, Santa Clara Valley Medical Center, San Jose, CA, United States; ^2^Beaumont Hospital-Trenton, Trenton, MI, United States; ^3^Department of Neurosurgery, Stanford University, Stanford, CA, United States; ^4^Division of Physical Medicine and Rehabilitation, Department of Neurosurgery, The University of New Mexico, Albuquerque, NM, United States; ^5^Division of Physical Medicine and Rehabilitation, Santa Clara Valley Medical Center, San Jose, CA, United States; ^6^Department of Orthopedic Surgery, Stanford University, Stanford, CA, United States

**Keywords:** head/cranial computed tomography scan (CT scan), axial injury, extra-axial injury, traumatic brain injury (TBI), burden of care, FIM® instrument (FIM)

## Abstract

**Background:** Head computed tomography (CT) scans are widely used in acute head injury for medical triage and surgical decisions, yet there are contradictions on the prognostic value of different head CT classifications. The intra-axial (axial) lesion vs. extra-axial lesion is a well-known systemic classification but has not been applied into clinical practice since there is no evidence-based support for its prognostic value.

**Hypothesis:** Axial injury is related to worse functional independence compared to extra-axial injury at admission to and discharge from acute rehabilitation hospitalization.

**Design:** Observational retrospective study.

**Settings and participants:** Data from 71 participants who were enrolled at an acute rehabilitation hospital in the Northern California Traumatic Brain Injury Model System of Care (NCTBIMS) between 2005 and 2018 were included in the analysis.

**Main outcome measure and statistical analysis:** Results of non-contrast head CT within the first 7 days after injury were analyzed to determine those with axial vs. extra-axial lesions. Functional Independence Measure (FIM) total scores were compared between the axial vs. extra-axial groups at admission and discharge using parametric and non-parametric tests.

**Results:** There were no statistically significant group differences in FIM total scores at rehabilitation admission and discharge between the axial group and extra-axial groups.

**Conclusion:** In this cohort of patients there was no evidence to support the hypothesis that axial injury is related to worse functional independence compared to extra-axial injury at rehabilitation admission and discharge. Utilizing MRI findings or other outcome measures, such as the 10 meter ambulation test or cognitive tests, may provide better sensitivity to potential functional differences.

## Introduction

Traumatic Brain Injury (TBI) presents a global health concern, as it has become one of the leading causes of death and disability worldwide (1). From 2006 to 2014, the total number of TBI-related emergency department (ED) visits, hospitalizations, and deaths increased by 53% in the United States (2). It was estimated that $21.4 billion was spent for TBI-related admissions and $8.2 billion for TBI-related ED visits in 2010 (3). Given the cognitive and physical challenges associated with TBI, patients with TBI often rely on caregivers for their daily living—half of whom experience elevated psychological distress from the increased burden of care (4). Even mild TBI has been shown to have long-term cognitive and emotional consequences that impede daily social and professional life (5). These consequences highlight the importance of TBI research to improve prognosis and treatment to maximize functional recovery.

Non-contrast head computed tomography (CT) scans have been used widely as a diagnostic tool in acute head injury because they are cost-effective, widely available, and detect hemorrhages with high sensitivity. Together with clinical assessment, Head CT scans are used in the classification of TBI severity, along with loss of consciousness duration, post-traumatic amnesia (PTA) duration, and the Glasgow Coma Scale (GCS) and to augment clinical assessment in medical triage and surgical management (6). For these reasons, characteristics of head CT scans have been used in the investigation of prognostic markers for individuals with TBI.

The most well-known head CT classification system is the Marshall head CT classification, which is based on the degree of midline shift, the status of the mesencephalic cisterns, and the presence or absence of surgical masses in predicting mortality (7). Maas et al. provided evidence for the prognostic value of the Marshall classification with traumatic subarachnoid hemorrhage (SAH), basal cisterns, and midline shift being strong predictors of GOS at 6 months (8). In combination suggests that the Marshall classification may provide predictive value to not only mortality but also functional outcomes. However, a recent study found that the Marshall classification does not inform functional prognosis or rehabilitation planning (9).

Beyond the Marshall classification, other investigators have examined prognosis of head CT scans by using different head CT features with varying results. Preliminary results by Rao et al. suggested that the classification for head CT lesion characteristics (normal/no lesions, one hemisphere lesion, and bilateral lesions) led to marked differences in rehabilitation outcomes, including transfers, dressing, and basic intellectual skills (10). However, this study did not include a statistical analysis. Using the GOS, Fearnside et al. found three CT predictors of mortality (the presence of cerebral edema, intraventricular hemorrhage, and the degree of midline shift) as well as three indicators of poor outcomes (SAH, intracerebral hematoma, and intracerebral contusion) (11). Wardlaw et al. found that the presence of SAH and the overall appearance of the CT scan are prognostic indicators for survival after TBI (12). Englander et al. suggested that the presence of either a midline shift >5 mm or a subcortical contusion on acute CT scans is associated with a greater need of assistance with ambulation, activities of daily living, and global supervision at rehabilitation discharge based on Functional Independence Measure (FIM) and Disability Rating Scale (DRS) measures (13).

Contrary to the previous studies, Brown et al. did not find CT scan characteristics, including the presence or absence of any pathology, the presence or absence of midline shift >5 mm, or the presence or absence of subcortical contusion, to predict FIM and DRS scores (14). Similarly, Sherer et al. did not find evidence of predictive value in early CT scan characteristics, such as the presence of abnormalities on CT scans, the total number of brain lesions, or the volume of lesions (15).

Another well-known head CT classification is the distinction between intra-axial (axial) vs. extra-axial lesions. Extra-axial lesions include epidural hematomas, subdural hematomas, and SAH (16), while axial lesions include all lesions beneath the pia mater, such as intra-parenchymal hemorrhage or contusion and intraventricular hemorrhage (17). As extra-axial lesions are often easier to access and treat than axial lesions, axial lesions are known to be more serious injuries (17). However, no studies have established differential outcomes after axial or extra-axial lesions for individuals with TBI; thus, this CT classification system has not been applied in clinical practice. Given the mixed findings of previous studies examining the prognostic value of CT scan classifications, this study was motivated to investigate the utility of (axial) and extra-axial lesions as an alternative systems-based approach.

Thus, the aim of this study was to identify the effect of axial and extra-axial lesions on the functional outcomes in patients with TBI. Specifically, this study tested the hypothesis that axial injury would be related to worse functional independence, compared to extra-axial injury, at admission to and discharge from acute rehabilitation hospitalization.

## Methods

### Participants

Participants enrolled in this retrospective study included individuals in the Northern California Traumatic Brain Injury Model System of Care (TBIMS) longitudinal study who were injured between May 2005 and August 2018. The inclusion criteria for patients in TBIMS include English and/or Spanish-speaking, with moderate to severe TBI (i.e., trauma-related head CT abnormalities, loss of consciousness > 30 min, or GCS in the ER < 13), presented to an acute care hospital within 72 h of injury, were at least 16 years old at the time of injury, and provided consent to participate in the TBIMS by the patient, family, or legal authorized representative (18).

**Figure 1:**
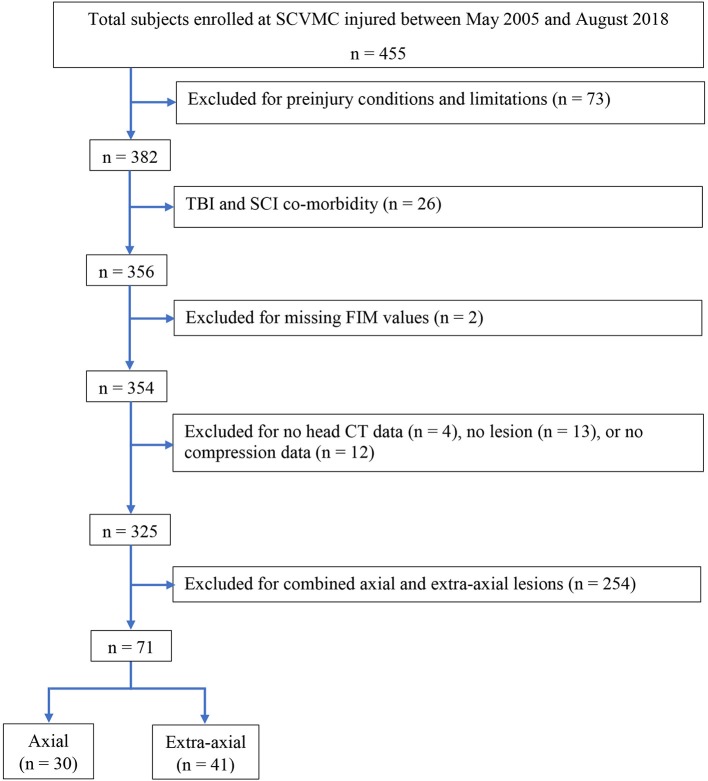
Flowchart of the study participants.

To limit potential confounding factors, patients were excluded from this study if they presented with long-lasting pre-injury conditions or limitations impeding their ability perform daily activities measured by FIM scores (i.e., physical, mental, or emotional conditions present for at least 6 months which limit a patient's ability for self-care, transportation, ability to learn, or work). Participants with spinal cord injury at the time of the injury were also excluded to avoid potential confounds. Individuals with mixed axial and extra-axial lesions were also excluded. The remaining 71 participants were included in this analysis (Figure 1), 30 of whom had axial lesions and 41 extra-axial lesions.

### Data Collection for Northern California Traumatic Brain Injury Model System of Care

The TBIMS provides a protocolized data abstraction certification process as well as regular data collector meetings and data checks to standardize data collection (https://www.tbindsc.org). Each research staff member received appropriate training and certification. During the participant's inpatient rehabilitation stay, interviews were conducted providing demographic and pre-injury history data. Research staff abstracted additional data from the participant's medical records according to the TBIMS protocol. Data collected at admission and discharge was used for this study.

### Measures

#### FIM Instrument

Functional Independence Measure (FIM) scores are often used in rehabilitation settings to quantify the level of disability of a patient (19, 20). The total FIM score ranges from 18 to 126 with a lower FIM score corresponding to greater assistance needed for the patient to perform simple, vital activities of daily living. The FIM assesses 13 motor and 5 cognitive functions for a total of 18 functions. Each function is on a 1 (total dependence) to 7 (total independence) scale. The motor subscale includes feeding, grooming, bathing, dressing the upper, and lower body, toileting, bladder and bowel management, bed to chair, or wheelchair transfers, toilet transfers, shower or tub transfers, and locomotion while the cognitive subscale includes comprehension, expression, social interaction, problem solving, and memory.

The sum of the cognitive and motor FIM scores provides the individual with a total FIM score that can be classified into one of seven levels: Total Assistance (18–30 FIM score) requiring more than 8 h of care per day, Maximal Assistance (31–53) requiring 6–7 h of care per day, Moderate Assistance (54–71) requiring 4-5 h of care per day, Minimal Assistance (72–89) requiring 2–3 h of care per day, Supervision or Set Up (90–107) requiring 1–2 h of care per day, Modified Independence (108–119) requiring <1 h of care per day, and Complete Independence (120–126) with no care needed per day (19).

#### Head CT Scan

Data was collected by research staff certified in TBIMS Form I CT Data coding (18). CT data was collected based on the combination of reports taken from radiographic non-contrast head CT results within the first 7 days of injury. The data was abstracted directly from the radiologist's CT impressions and findings. Variables from the following categories are abstracted from radiology reports: extent of intracranial compression, intracranial (both axial and extra-axial) hemorrhage and/or contusions, and intraparenchymal fragments; all but intraparenchymal fragments were considered in our analyses. The *Introduction to Diagnostic Radiology* provides examples of the different CT parameters used in this study (16).

As previously mentioned, extra-axial lesions include epidural hematoma, subdural hematoma, and SAH, while axial lesions include punctate hemorrhage, intraventricular hemorrhage, and cortical or non-cortical parenchymal contusions or hemorrhage. Intracranial compression severity was classified into four groups: (1) shift >5 mm or herniation, (2) cistern compression with shift < 5 mm, (3) without cistern compression and shift 1–5 mm, and 4) no shift or shift < 1 mm (18).

### Study Design

Data in this observational retrospective study followed a repeated measures design.

### Statistical Analysis

#### Participants Characteristics

Demographic variables (age, acute length of stay, rehabilitation length of stay, sex, and ethnicity) were tested for group differences using a *t*-test, chi-square test, or Fisher's exact test. In addition, we also identified the impact of intracranial compression severity on FIM total scores at these time points. Time from injury to an acute setting was not assessed because only 2 participants were not admitted to an acute hospital on the same day as their injury; one participant was admitted on the next day and another was admitted 3 days after their injury.

#### The Impact of Axial vs. Extra-Axial on FIM Total Scores From Rehabilitation Admission to Discharge

Seventy-one subjects were included in these analyses. First, a repeated measures ANOVA was used to test for group effect (axial vs. extra-axial lesion), time effect (admission vs. discharge), and group x time interactions. Due to a violation of normality in the FIM discharge data, analogous non-parametric tests were also employed. Mann-Whitney *U* tests were carried out to compare FIM data at admission and discharge and a difference score (discharge FIM- admission FIM) was created and tested using a Wilcoxon signed rank test to assess group differences in the FIM changes. Parametric and non-parametric results are presented.

## Results

### Participant Characteristics

Characteristics of the 71 participants are presented in Table 1. Differences between groups by sex, acute length of stay, rehabilitation length of stay, age, ethnicity, and intracranial compression severity were examined. There were no significant group differences in sex, acute and rehabilitation length of stay, and ethnicity (*p* > 0.1). Although individuals in the extra-axial group had a significantly greater proportion of individuals with cistern compression with a midline shift of >5 mm, compared to the axial group (Fisher's exact test *p* < 0.001), the Kruskal Wallis test did not reveal a significant association between CT compression grouping and FIM scores [FIM admission: $\chi_{\left(3\right)}^{2}$ = 0.448, *p* = 0.93; FIM discharge: $\chi_{\left(3\right)}^{2}$ = 2.4, *p* = 0.46).

**Table 1:** Participant characteristics (age, sex, ethnicity, and head CT compression characteristics) by extra-axial vs. axial lesion.

**Characteristics**		**Extra axial** **(*N* = 41)**	**Axial** **(*N* = 30)**	**Difference test**	
				**t/χ^2^**	***p*-value**
Age					
Mean (SD)		32.8 (16.5)	30.3 (16.7)	0.36	0.72
Acute LOS		19.0 (10.7)	14.9 (9.6)	1.7	0.10
Mean (SD)					
Rehabilitation LOS					
Mean (SD)		22.2 (18.9)	16.3 (10.3)	1.5	0.13
Sex (%)	Female	39.6	26.7	1.7	0.20
	Male	60.4	73.3		
Ethnicity (%)	White	57.7	60.0	**	0.99
	Black	7.0	6.7		
	Asian/Pacific Islander	5.6	6.7		
	Hispanic	25.4	23.3		
	other	4.2	3.3		
CT compression (%)	No visible intracranial compression	68.8	96.7	**	<0.01
	Cisterns are present but midline shift 1–5 mm	8.3	0.0		
	Cisterns compressed or absent with midline shift 0–5	4.2	3.3		
	Midline shift > 5 mm or herniation	18.8	0.0		

Age is represented in years. **Fisher's exact test.

### The Impact of Axial vs. Extra-Axial on FIM Total Scores From Rehabilitation Admission to Discharge

**Figure 2:**
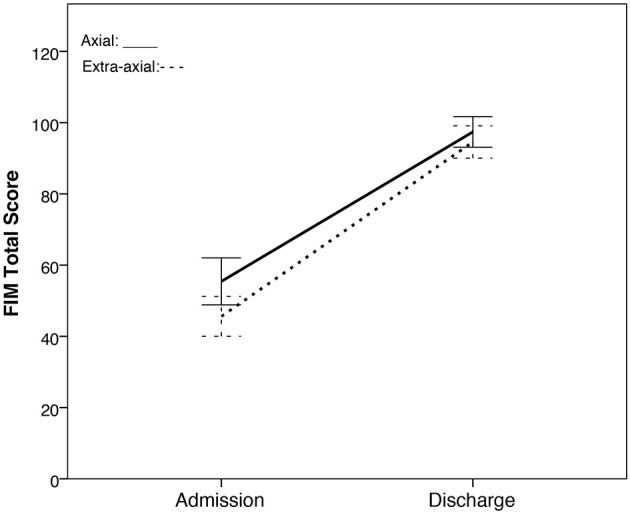
FIM total scores of the axial group vs. extra-axial group across two time points (rehabilitation admission and discharge). The axial group is represented by the solid line while the extra-axial group is represented by a dashed line. 95% confidence intervals are represented in the error bars.

The repeated measures ANOVA (rmANOVA) for the two groups (axial vs. extra-axial) x two time points (admission and discharge) on the FIM total score showed no main effect of group [*F*_(1, 69)_ = 1.969, *p* = 0.165, η^2^ = 0.028] but revealed a significant time effect [*F*_(1, 69)_ = 674.833, *p* < 0.001, η^2^ = 0.907]. A significant group x time interaction was not observed [*F*_(1, 69)_ = 3.363, *p* = 0.071, η^2^ = 0.046; Figure 2].

Due to the violation in the Shapiro-Wilk Test of normality for the FIM total score of the extra-axial group at discharge [W_(41)_ = 0.938, *p* = 0.026], non-parametric tests were conducted. In line with rmANOVA, Mann-Whitney *U* tests showed no significant group differences in the distribution of FIM total score at admission (*U* = 447.5, *Z* = −1.951, *p* = 0.051) and discharge (*U* = 588, *Z* = −0.314, *p* = 0.753) as well as between FIM changes from admission to discharge (*U* = 456.5, *Z* = −1.846, *p* = 0.065). Wilcoxon signed rank test reveals significant within group differences in the median of FIM total scores between admission and discharge for both extra-axial (*Z* = −5.579, *p* < 0.001) and axial (*Z* = −4.784, *p* < 0.001) groups.

## Discussion

This study is one of the first to investigate the effect of axial and extra-axial lesions on functional independence at admission and discharge from an acute rehabilitation center. The study did not support the hypothesis that axial injuries relate to worse functional outcomes than extra-axial injuries. Although mean FIM total scores for participants with axial injuries showed greater functional independence than those with extra-axial injuries in this cohort of patients, there was no statistically significant difference. Similar to Brown et al.'s studies and Sherer et al.'s, this study was not able to illustrate the predictive value of head CT scans (9, 14, 15). More work needs to be done to understand how CT features, or other imaging modalities, may be related function outcomes in the acute phase of injury.

In this study, there were significant FIM total score improvements within each group. The extra-axial lesion group tended to have lower FIM total scores at admission (*p* = 0.051), which may represent slightly greater injury severity in the extra-axial group compared to the axial group. Alternatively, the individual with axial lesions that were included in this study survived and were authorized to receive rehabilitation, which may represent a higher functioning group. These differences in average FIM scores did not represent categorical differences in levels of assistance needed since both the axial and extra-axial groups were categorized as maximal assistance (6–7 h of care per day) at admission. The greater change from admission to discharge FIM total score in the extra-axial lesion group compared to the axial lesion group approached, but did not reach, statistical significance (rmANOVA interaction, *p* = 0.071, MWU test on FIM change, *p* = 0.065) and, similar to admission FIM totals, both groups recovered to the same functional independence level (supervision/setup; 1–2 h of care per day) at discharge. Given the trend toward significance, a larger sample size may be needed to examine whether individuals with extra-axial lesions compared to axial lesions have greater functional improvements during their rehabilitation stays.

Significant differences in the degree of compression were observed between the two groups, although there was no significant effect of degree of compression on FIM total scores at admission or discharge (Table 2). Similar to our findings, previous work using the Marshall classification did not find degree of compression to inform functional prognosis and rehabilitation planning after TBI (9). It is important to note that although we did not observe compression effects on FIM total score, other functional measures may reveal differences in the impact of intracranial compression.

**Table 2:** Summary of group averages and standard deviations by CT compression grouping.

**CT grouping**	***N***	**Admission** **mean (SD)**	**Discharge** **mean (SD)**
No visible intracranial compression	57	51.8 (19.8)	97.0 (13.1)
Cisterns are present but midline shift 1-5 mm	3	52.7 (13.6)	98.7 (5.1)
Cisterns compressed or absent with midline shift 0–5	2	45.0 (14.1)	91.5 (7.78)
Midline shift > 5 mm or herniation	9	43.7 (21.0)	92.2 (21.5)

*The number of participants included (N), means, and standard deviations (SD) are included for each CT compression grouping*.

*No significant differences in FIM total score at admission or discharge were observed across CT compression groups [FIM admission: $\chi_{\left(3\right)}^{2}$ = 0.448, p = 0.93; FIM discharge: $\chi_{\left(3\right)}^{2}$ = 2.4, p = 0.46]*.

The current study has several limitations that are worthy of discussion. Although data collection followed the TBIMS protocol, which included certification for CT medical record data abstraction and coding, and the authors made considerable efforts to exclude and account for certain potential confounds, there may still be some unforeseen confounds. For instance, only patients who survived to rehabilitation discharge were included into the study. In addition, to exclude confounds, many subjects were excluded reducing our initially large sample to a relatively small sample size. As the TBIMS is longitudinal and accepts patients transferred from other healthcare facilities, there may be multi-rater bias from different radiologists who read head CTs. Although the FIM instrument is a widely used functional assessment in the rehabilitation community, alternative functional outcome measures (10 meter ambulation test or cognitive tests) may be more sensitive to differences between individuals with axial and extra-axial lesions; future studies should consider alternative measures and larger sample sizes. We also did not extend our study to the long-term follow-up after discharge from acute rehabilitation, which may be an interesting extension for future work. Taken together, more studies, during both the acute and long-term phase, are necessary to investigate the prognostic value of this head CT classification.

In conclusion, this study does not support the hypothesis that axial injury relates to worse functional outcomes in the acute rehabilitation setting when compared to extra-axial injury after TBI. The study was not able to define group differences in functional outcomes using a well-known classification. More studies with larger sample sizes are needed to better examine the impact of CT findings during acute rehabilitation phase as well as long-term outcomes which may inform clinical care.

## Data Availability Statement

The raw data supporting the conclusions of this manuscript will be made available by the authors, without undue reservations, to any qualified researcher.

## Ethics Statement

The studies involving human participants were reviewed and approved by Santa Clara Valley Medical Center Institutional Review Board. The patients/participants provided their written informed consent to participate in this study.

## Author Contributions

TD is the last author, directed the project, assisted in data interpretation, and edited the manuscript. NQ, JD, BD, and RE analyzed data and wrote most parts of the paper. EP assisted in reviewing and editing the manuscript. AA assisted in presenting results into tables.

### Conflict of Interest

The authors declare that the research was conducted in the absence of any commercial or financial relationships that could be construed as a potential conflict of interest.
